# Prevalence and clinical characteristics of X-linked hypophosphatemia in Paraná, southern Brazil

**DOI:** 10.20945/2359-3997000000296

**Published:** 2020-10-09

**Authors:** Carolina Aguiar Moreira, Tatiana M. R. Lemos Costa, Julia Vieira Oberger Marques, Lucimary Sylvestre, Ana Cristina R. Almeida, Eliane M. C. P. Maluf, Victória Z. C. Borba

**Affiliations:** 1 Universidade Federal do Paraná Hospital de Clínicas Divisão de Endocrinologia (SEMPR) Curitiba PR Brasil Divisão de Endocrinologia (SEMPR), Hospital de Clínicas, Universidade Federal do Paraná, Curitiba, PR, Brasil; 2 Fundação Pró-Renal Seção de Histomorfometria Óssea Lab PRO Curitiba PR Brasil Lab PRO, Seção de Histomorfometria Óssea, Fundação Pró-Renal, Curitiba, PR, Brasil; 3 Hospital Pequeno Príncipe Serviço de Nefrologia Pediátrica Curitiba PR Brasil Serviço de Nefrologia Pediátrica, Hospital Pequeno Príncipe, Curitiba, PR, Brasil; 4 Pontifícia Universidade Católica do Paraná Escola de Medicina Curitiba PR Brasil Escola de Medicina, Pontifícia Universidade Católica do Paraná, Curitiba, PR, Brasil; 5 Secretaria de Saúde do Paraná Curitiba PR Brasil Secretaria de Saúde do Paraná, Curitiba, PR, Brasil; 6 Universidade Positivo Curitiba PR Brasil Universidade Positivo, Curitiba, PR, Brasil; 7 Universidade Federal do Paraná Programa de Pós-Graduação Curitiba PR Brasil Programa de Pós-Graduação, Universidade Federal do Paraná, Curitiba, PR, Brasil

**Keywords:** Rickets, hypophosphatemia, fibroblast growth factor 23, human, X-linked hypophosphatemia, PHEX mutation

## Abstract

**Objective:**

The aim of this cross-sectional study was to estimate the prevalence of XLH in Paraná, a state in southern Brazil, and report the clinical features and complications of the disease.

**Materials and methods:**

We invited all endocrinologists (n = 205), nephrologists (n = 221), orthopedic surgeons (n = 1020), and pediatricians (n = 1000) in Paraná to fill out an electronic survey with information on patients with X-linked hypophosphatemia (XLH), and searched the records of the state's health department for all calcitriol prescriptions in 2018.

**Results:**

In all, 244 (10%) specialists responded to the email, of whom 18 (7.4%) reported to be taking care of patients with XLH and answered the online survey. A total of 57 patients with XLH were identified (prevalence 5 per million inhabitants). The median age at diagnosis was 22 years, and 42.2% were children and adolescents. Fifteen patients had genetic testing showing a *PHEX* mutation. Overall, 91.2% had bone deformities, 30.8% had a history of fragility fractures, and 22.4% had renal complications.

**Conclusion:**

This study demonstrated a prevalence of XLH of 5 cases per million inhabitants in the state of Paraná, a rate lower than the one reported in other countries. Manifestations of renal calcification and bone fragility were frequent among the patients. This is the first epidemiological study evaluating the prevalence and clinical presentation of XLH in Latin America.

## INTRODUCTION

X-linked hypophosphatemia (XLH) is a lifelong condition associated with decreased serum phosphate levels and clinically characterized by rickets in children and osteomalacia in adults ( 1 ). It is the most common type of hereditary hypophosphatemia and was first described by Albright in 1937 ( 2 ).

XLH is caused by a loss-of-function mutation in the *PHEX* (phosphate regulating endopeptidase homolog, X-linked) gene, leading to increased circulating levels of fibroblast growth factor 23 (FGF23) ( 3 , 4 ) and consequent phosphaturia, hypophosphatemia, and low 1,25-dihydroxyvitamin D levels ( 5 , 6 ). Clinically, these laboratory abnormalities lead to defective bone mineralization, manifesting in most patients as bone pain, lower limb deformity, growth impairment, and physical dysfunction ( 7 , 8 ). These manifestations start early in life and remain throughout adulthood as chronic bone complications affecting the patient's quality of life ( 9 ). In fact, a recent survey including adult patients with XLH has shown that nearly all affected individuals present bone or joint stiffness, while 44% have a history of fractures, demonstrating a substantial disease burden ( 10 , 11 ).

Burosumab, an antibody against FGF23, has recently emerged as a novel therapy for children and adults with XLH ( 12 , 13 ). Still, most patients with XLH in Brazil continue to receive conventional therapy consisting of multiple daily doses of oral phosphate salts and vitamin D metabolites or analogues as replacement therapy, a treatment that has been associated with long-term complications related to calcification, including kidney calcification leading to impaired renal function ( 14 ).

Few data are available on the prevalence of XLH in the general population worldwide. The prevalence of XLH in some European countries has been reported to range between 16 to 48 cases per million individuals, although these rates are mostly related to children and adolescents ( 15 – 17 ). To our knowledge, no studies have been conducted to assess the epidemiology of XLH in Latin America.

Based on these considerations, we conducted the first study to estimate the prevalence of XLH in a defined geographic region in Latin America, encompassing the entire state of Paraná, located in southern Brazil. A secondary objective of the study was to evaluate in more detail the clinical and biochemical characteristics of a subcohort of children and adults with XLH following up in a bone unit at a tertiary hospital located in Curitiba, the capital of the state of Paraná and the largest city in the state.

## MATERIALS AND METHODS

This cross-sectional study was approved by the Ethics Committee at *Hospital de Clínicas, Universidade Federal do Paraná* (Curitiba, Paraná) and conducted according to the Declaration of Helsinki. Written informed consent was signed by all patients prior to inclusion in the study (CAAE Hospital de Clínicas: 85053618.0.3001.0097; Hospital Pequeno Príncipe: 85053618.0.00000096).

### Survey and active search cohort

We performed an active search of all cases of XLH in the state of Paraná, Brazil, during the year of 2018. The diagnostic criteria were clinical and laboratory features of XLH, such as short stature and bone deformities along with hypophosphatemia and hyperphosphaturia (tubular maximum reabsorption rate of phosphate [TMP/GFR] > 85%). A genetic test showing *PHEX* mutation was also a diagnostic criterion for XLH. We invited (via email or phone calls) to participate in the survey all physicians living in Paraná and working in specialties involved in the care of patients with XLH ( *i.e.* , endocrinologists, pediatricians, orthopedic surgeons, and nephrologists). A total of 205 endocrinologists, 1000 pediatricians, 1020 orthopedic surgeons, and 221 nephrologists were contacted by mail, sent by each specialty society, and received an invitation to participate in the survey. A reminder was sent to those specialists following up patients with XLH, including a request to respond to our survey. Physicians who responded indicating that they took care of patients with XLH were asked to complete a standardized online survey providing clinical data of their patients. The survey collected the following information about the patient: city of birth, age at data collection and at diagnosis, family history of XLH, disease duration, clinical signs and symptoms at data collection, current and past treatment, presence or absence of renal complications, and presence or absence of genetic testing to confirm mutation of the *PHEX* gene.

To ensure that the study included all XLH patients in the state of Paraná, we also performed an active search of calcitriol prescriptions dispensed by the high-cost drug supply sector of Paraná's health department (CEMEPAR). CEMEPAR provides free-of-charge calcitriol, which is used by most patients with XLH. In this search, we obtained a list of patients’ records and the names of the physicians in charge of the patients receiving the medication and whose International Disease Code (ICD) corresponded to hypophosphatemic rickets. No laboratory or chart review was conducted in the overall cohort, and all information was obtained from the physicians taking care of the patients.

Physicians identified through this search who had not yet responded to the email were also invited by telephone to participate in the survey and complete the survey with the patients’ clinical data. Patients who had already been included in the survey were not included again.

To calculate the prevalence of XLH in the state of Paraná, we divided the number of cases of XLH found in the year 2018 by the estimated total number of inhabitants in the state of Paraná in the same year (11,348,937).

### Outpatient subcohort following up in a bone unit at a tertiary hospital

We searched the medical records of patients with hypophosphatemia following up at our bone unit at the Endocrine Division in *Hospital de Clínicas* at *Universidade Federal do Paraná* (SEMPR) to obtain data on clinical characteristics, current and prior treatment, and laboratory test results.

Of note, all patients in this subcohort who had a clinical or genetic diagnosis of XLH were also included in the overall cohort. In addition to collecting similar data as those collected from the patients identified through the survey, we also searched the medical records of these patients for results of renal ultrasound and bone densitometry.

The *PHEX* gene mutation was evaluated in 7 patients in this subcohort according to the following technique: genomic DNA was collected in an oral swab. The *PHEX* gene (OMIM * 300550) exons were captured with Agilent Mendelics Custom Panel V3, followed by next-generation sequencing with an Illumina HiSeq sequencer (Mendelics Análise Genômica S. A., São Paulo, SP, Brazil). Sequence alignment and identification of variants followed bioinformatics protocols, using the version GRCh37 of the human genome as a reference.

### Statistical analysis

The collected data were unidentified, organized into Google Docs spreadsheets, and analyzed with SPSS 22.0 (Statistical Package for Social Sciences, SPSS Inc., Chicago, IL, USA).

To estimate the prevalence of XLH, we considered the cases related to the year 2018 identified by the active search in relation to the entire population of the state of Paraná estimated for that same year, obtained from the Brazilian Institute of Geography and Statistics (IBGE) ( 18 ).

To analyze associations between variables, we grouped all variables into categories and applied the chi-square test or Fisher's exact test. Continuous variables are presented as mean ± standard deviation or median (range), and categorical variables as frequency and percentages. The significance level was considered at p < 0.05.

## RESULTS

### Survey and active search cohort

**Figure 1:**
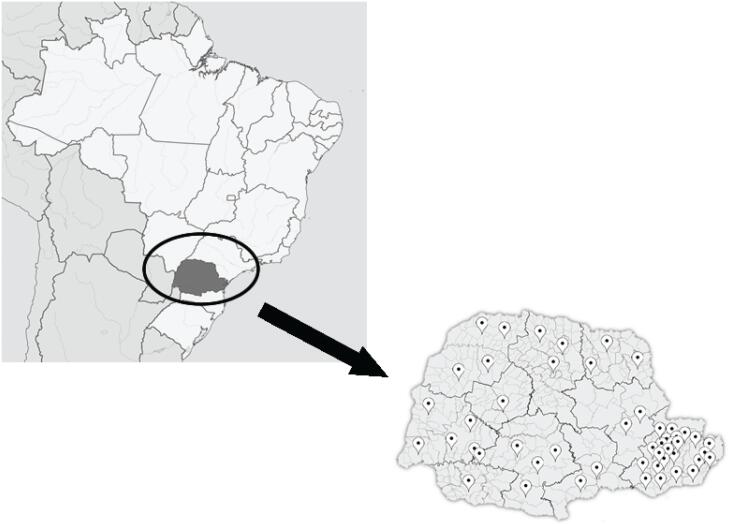
Geographic distribution of the patients with XLH across the state of Paraná.

Of all specialists contacted, 244 (10%) responded to the survey email, and from these, 18 (7.4%) reported to be taking care of patients with XLH and answered the online survey. These specialists belonged to six different medical specialties, comprising mostly adult and pediatric endocrinologists (68%), followed by pediatricians, adult and pediatric nephrologists, and orthopedic surgeons. Three patients were identified from the CEMEPAR active search. Overall, the survey identified 57 children and adults with clinical and laboratory diagnosis of XLH in the state of Paraná during 2018 (prevalence of 5 cases per million inhabitants). A subanalysis including only individuals aged 19 years or below yielded a prevalence of 7.8 cases per million inhabitants aged up to 19 years. Figure 1 shows the geographic distribution of the patients across the state of Paraná.

Table 1 summarizes the information reported by the physicians related to the 57 patients with XLH. The median age of the patients at data collection was 22.0 years (range 1–55 years), and 42.2% were younger than 18 years. Overall, 60.5% were female and 65.4% had a family history of XLH.

**Table 1:** Clinical characteristics of 57 patients with X-linked hypophosphatemia (XLH) identified in 2018 in the state of Paraná, Brazil

Clinical characteristics		Values *
Sex		
	Female	23 (40.4%)
	Male	15 (26.3%)
	Not informed	19 (33.3%)
Age at data collection (groups)		
	0 to 3 years	4 (7.0%)
	4 to 10 years	9 (15.8%)
	11 to 20 years	13 (22.8%)
	21 to 30 years	22 (38.6%)
	≥ 31 years	9 (15.8%)
Median age at data collection (range)		22 years (1 – 55 years)
Median time since diagnosis (range)		15 years (6 months – 45 years)
Family history positive for XLH		34 (59.64%)
Lower limb deformity		52 (91.2%)
Fragility fractures		16 (28.1%)
Renal complications		11 (19.3%)
	Nephrocalcinosis	7
	Nephrolithiasis	3
	Membranous nephropathy	1

* Number of patients (range or percentage in relation to the entire cohort).

The median time since diagnosis was 15 years (range 6 months to 45 years) and was above 20 years in 36.8% of the cases. In two (5%) patients, the diagnosis was established less than 1 year before the data collection. The diagnosis of XLH in most patients was based on clinical manifestations and laboratory hypophosphatemia. Fifteen patients had a genetic diagnosis with evidence of *PHEX* mutation.

### Clinical characteristics

Lower limb deformity was the most common clinical manifestation, present in 52 (91.2%) cases, and showed no association with family history of XLH (yes or no) or disease duration (< 10 or ≥ 10 years; Fisher's exact test p=0.51).

Fragility fractures were reported in 16 patients (30.8%) and were more frequent in patients with disease duration longer than 10 years (n = 13) compared with those in whom the duration of the disease was below 10 years (n = 3; 81.3% vs. 18.8%, respectively, p = 0.007). The history of fracture was not related to the presence of family history of XLH (p=0.07). Chronic renal complications were identified in 11 (19.3%) patients and included nephrocalcinosis (n = 7), nephrolithiasis (n = 3), and membranous nephropathy (n = 1). The presence of renal calcification seemed to have no association with disease duration, positive family history of XLH, or history of fractures, although the number of patients with renal complications was too small for statistical analysis.

Concerning treatment, 90% of the patients received some type of conventional medication, most frequently a combination of phosphate salts and calcitriol (43%), a combination of calcium, phosphate salts, and calcitriol (17.3%), or calcitriol alone (15.4%). None of the patients used burosumab.

### Outpatient subcohort following up in the bone unit at SEMPR

We identified 22 patients with hypophosphatemia. Three of these patients were excluded due to other causes of hypophosphatemia, including Fanconi syndrome and mucoviscidosis. All 19 remaining patients had a clinical and radiological diagnosis of XLH and were also part of the original cohort, including 7 (36.8%) with genetic confirmation of *PHEX* mutation. The details of the genetic analysis are shown in Table 2 .

**Table 2:** Results of genetic tests obtained from seven patients with X-linked hypophosphatemia (XLH) in the outpatient subcohort

Patient *	Mutation	Mutation description	Zygosis
1	c.2057T>A (p.Leu686Gln	Novel missense mutation	Heterozygous
2	c.2057T>A (p.Leu686Gln	Novel missense mutation	Heterozygous
3	c.2057T>A (p.Leu686Gln	Novel missense mutation	Homozygous
4	c.266.059 C > T p.Arg747	Definitely pathogenic	Heterozygous
5	c.244.618 C > A p.Ala653Asp	Probably pathogenic	Heterozygous
6	c.237.176 G > A p.Gly575Glu	Probably pathogenic	Heterozygous
7	c.237.176 G > A p.Gly575Glu	Probably pathogenic	Heterozygous

* Patients 1, 2, and 3 are two sisters and their father. Patients 6 and 7 are mother and daughter.

The analysis of the *PHEX* gene in two sibling sisters and their father was positive for a novel missense mutation (variant of uncertain significance). In one patient, the analysis of the gene showed a definitely pathogenic mutation, and in three other patients, a probably pathogenic mutations. Table 2 shows the main results of the genetic analysis of these patients.

In this outpatient subcohort, 14 (87.0%) patients presented bone pain, and 9 (56.6%) had a history of fragility fractures affecting mostly the femur (n = 5). A history of orthopedic surgery was present in 9 (47.4%) patients.

Of 12 patients who underwent renal ultrasound, renal complications were observed in 4 (33.3%), including 1 case each of nephrolithiasis, nephrocalcinosis, hydronephrosis, and membranous nephropathy. During follow-up, one patient presented secondary hyperparathyroidism after treatment ( Table 3 ).

**Table 3:** Clinical characteristics of 19 patients with X-linked hypophosphatemia (XLH) in the outpatient subcohort

Clinical characteristics		Values *
Sex		
	Female	13 (68.4%)
	Male	6 (31.6%)
Age		
	0 to 3 years	9 (47.4%)
	4 to 10 years	5 (26.3%)
	11 to 20 years	1 (5.3%)
	21 to 30 years	2 (10.5%)
	≥ 31 years	2 (10.5%)
Age at diagnosis – mean (range)		10.7 years (1.1 – 49.0 years)
Follow-up duration – mean±SD		126.5 ± 89.8 months
Family history positive for XLH		9 (47.4%)
Lumbar spine deformity		7 (36.8%)
Cranial deformity		3 (15.8%)
Epiphyseal enlargement		10 (52.6%)
Bone pain		15 (78.9%)
Teeth abnormalities (except for cavities)		5 (26.3%)
Lower limb deformity		17 (89.5%)
Fragility fractures		9 (47.4%)
	Femoral	5
	Others	4
Orthopedic surgery (osteotomy)		9 (47.4%)
Renal complications		4 (33.3%)
	Nephrolithiasis	1
	Nephrocalcinosis	1
	Hydronephrosis	1
	Membranous nephropathy	1

* Number of patients (range or percentage in relation to the entire cohort).

Eight patients (42.1%) underwent bone densitometry at the beginning of follow-up and presented a mean lumbar spine z-score of -2.24 and a total femur z-score of -2.20. All these patients progressed with bone mass gain once conventional treatment was started.

The median values of the main laboratory tests available in the patients’ medical records at treatment start were: total calcium 9.2 mg/dL (range 8.3–9.9 mg/dL), phosphorus 2.2 mg/dL (range 1.3–3.0 mg/dL), alkaline phosphatase 786.00 U/L (range 19–2109 U/L), PTH 62.5 pg/mL (range 30–523 pg/dL), and 25-hydroxyvitamin D 27.4 ng/mL (range 21–47 ng/mL).

The initial treatment in this subcohort was based on phosphate replacement in 9 patients (47%; mean daily dose 1.7 g [range 0.6–3.0 g]), calcitriol in 10 patients (52.6%; mean daily dose 0.32 μg [range 0.125–0.5 μg]), and calcium carbonate (daily dose 1000 mg) in 12 patients (63.2%).

## DISCUSSION

This first epidemiological Latin American study assessing the prevalence of XLH showed that the disease affects about 5 children and adults per million inhabitants in the state of Paraná, Brazil. This prevalence is low compared with the rates reported in other studies from Denmark, Japan, and Norway, which range from 16 to 48 per million inhabitants ( 15 , 17 , 19 ). In a recent study from Italy, also based on a survey filled out by physicians from 10 specialized centers, the prevalence rate of XLH among patients aged 0 to 17 years ranged from 19.9 – 50.1 cases per million ( 20 ). Our study also found a lower prevalence than this in a subanalysis restricted to individuals aged 19 years or below (7.8 cases per million). Of note, the median age at diagnosis in our study was 22 years. Prevalence studies of XLH including only adult cases are not available in the literature.

The study from Norway was the only one to confirm the *PHEX* mutation by genetic analysis in all 21 subjects evaluated, identifying 13 different pedigrees ( 16 ). The prevalence of XLH in that study (16 per million) was also lower than that in other studies. The authors suggested the possibility that female patients may have been underdiagnosed in their study since other XLH studies have reported a low penetrance of skeletal manifestations in female family members, while these manifestations are present in all male patients. This could also explain the lower overall prevalence found in our study compared with others. It is important to point out that two of our patients were older than 40 years and had been recently diagnosed with XLH. This delayed diagnosis confirms that not all physicians are familiar with the diagnosis of XLH, probably because of the subtle manifestations of the disease in some patients.

Since all other studies assessing the prevalence of XLH are limited to children and adolescents, the median age at the present study (which also included adults) was higher than the one reported in other studies. Notably, 13.8% of our patients were older than 41 years.

We found a high prevalence of renal complications associated with calcification, which affected half of our patients; specifically, nephrocalcinosis was more frequent than nephrolithiasis, a finding also reported in other recent studies in patients with XLH ( 8 , 13 , 14 ). The physiopathology behind the occurrence of renal calcification and secondary hyperparathyroidism in XLH is probably related to the long-term use of conventional therapy, in which repeated use of phosphate throughout the day and variation in serum phosphate increase renal phosphorus load ( 14 , 21 ). In fact, a study in children with XLH and nephrocalcinosis has suggested that the occurrence of nephrocalcinosis was associated with the dose of phosphate received ( 21 ). In contrast, another study found that nephrocalcinosis correlated with hypercalciuria and dose of calcitriol ( 22 ). In addition to the association of nephrocalcinosis with conventional treatment, the disease itself may lead to renal calcification, since hyperphosphaturia is an independent risk factor for the development of this condition ( 14 , 23 ). Furthermore, in a cohort of children and adults with XLH, nephrocalcinosis was found in 38.4% of the patients, while hypocitraturia, which is also considered a risk factor for renal calcification, was present in 28.2% of the cases ( 14 ). A similar prevalence of nephrocalcinosis was observed in the Italian study mentioned above ( 20 ). The increased finding of renal complications in our patients and in the literature calls attention to the importance of obtaining a renal ultrasound during follow-up of patients with XLH in search of early detection of renal calcification.

Bone fragility is frequent among patients with hypophosphatemic rickets of longer duration and may be secondary to osteomalacia caused by low levels of phosphate and 1,25-hydroxyvitamin D. Studies also report a high rate of skeletal involvement including fractures and history of osteotomy during childhood and adolescence ( 8 ). In addition to fractures, our cohort also had a high prevalence of bone pain and a history of osteotomy, reinforcing the evidence that the skeleton is profoundly affected by the disease. Indeed, a high rate of fractures and pseudo-fractures has been reported in a clinical trial of burosumab in XLH adults, in which the mean age of the patients was 40 years ( 13 ). Increased biochemical markers of bone remodeling and sclerostin levels have been recently reported in 27 adults with XLH regardless of treatment ( 24 ). Still, more studies are needed to elucidate the role of high bone turnover in the development of fractures. Other musculoskeletal abnormalities observed in XLH include osteoarthritis, enthesopathy, and impaired physical functioning ( 24 ). These findings suggest that long-term hypophosphatemia and low 1,25 dihydroxyvitamin D levels are critical for skeletal health.

Although our survey attempted to include all patients with XLH in the state of Paraná by targeting different aspects of their treatment (physicians in specialties potentially treating patients with XLH, dispensing of calcitriol prescriptions), a potential limitation of the study is the possibility of missed cases from specialists who may not have responded to our survey. An organized system of registration of rare diseases (similar to the one implemented in some European countries, allowing for more efficient retrieval of data) is unavailable in Brazil. Although our attempt to identify patients with XLH by active calling all specialists may not have ensured full coverage of all cases in the state, we have personally contacted by phone and/or email all specialists in the state potentially caring for these patients, increasing the odds of including all patients with XLH from the state of Paraná. Our search only included the database of calcitriol supplied by the government, although it is still possible that patients paying out-of-pocket for the medication may have been missed. Additionally, some patients – mainly adults – may no longer be receiving calcitriol, while others may be undiagnosed. Another potential limitation of our study is the absence of data related to the patients’ growth in height in the survey; thus, the prevalence and magnitude of growth impairment in our cohort are unknown. A major strength of our study is the inclusion of a well-defined geographical area in Brazil, increasing the possibility that our findings may reflect the overall prevalence of XLH in the entire country.

In conclusion, in the state of Paraná, the prevalence of XLH among children and adults was 5 per million inhabitants, a rate lower than the one reported in similar studies conducted in other countries. A high percentage of patients in our study had renal complications and bone fragility. This is the first epidemiological study assessing the prevalence and clinical manifestations of XLH in Brazil and Latin America. A registry of all cases of XLH and other rare diseases, following the model of certain European countries, is an important shortcoming of the Brazilian health care system that deserves more attention. This strategy would improve the collection of epidemiological data on rare diseases in our country.

## References

[1] Carpenter TO, Imel EA, Holm IA, Jan de Beur SM, Insogna KL. A clinician's guide to X-linked hypophosphatemia [published correction appears in J Bone Miner Res. 2015 Feb;30(2):394]. J Bone Miner Res. 2011;26(7):1381-8. 10.1002/jbmr.340 PMC3157040 21538511

[2] Albright F, Butler AM, Bloomberg E. Rickets resistant to vitamin D therapy. Am J Dis Child. 1937;54(3):529-47.

[3] Holm IA, Nelson AE, Robinson BG, Mason RS, Marsh DJ, Cowell CT, et al. Mutational analysis and genotype-phenotype correlation of the PHEX gene in X-linked hypophosphatemic rickets. J Clin Endocrinol Metab. 2001;86(8):3889-99. 10.1210/jcem.86.8.7761 11502829

[4] Drezner MK. PHEX gene and hypophosphatemia. Kidney Int. 2000;57(1):9-18. 10.1046/j.1523-1755.2000.00807.x 10620182

[5] Shimada T, Hasegawa H, Yamazaki Y, Muto T, Hino R, Takeuchi Y, et al. FGF-23 is a potent regulator of vitamin D metabolism and phosphate homeostasis. J Bone Miner Res. 2004;19(3):429-35. 10.1359/JBMR.0301264 15040831

[6] Shimada T, Kakitani M, Yamazaki Y, Hasegawa H, Takeuchi Y, Fujita T, et al. Targeted ablation of Fgf23 demonstrates an essential physiological role of FGF23 in phosphate and vitamin D metabolism. J Clin Invest. 2004;113(4):561-8. 10.1172/JCI19081 PMC338262 14966565

[7] Reid IR, Hardy DC, Murphy WA, Teitelbaum SL, Bergfeld MA, Whyte MP. X-linked hypophosphatemia: a clinical, biochemical, and histopathologic assessment of morbidity in adults. Medicine (Baltimore). 1989;68(6):336-52. 2811660

[8] Chesher D, Oddy M, Darbar U, Sayal P, Casey A, Ryan A, et al. Outcome of adult patients with X-linked hypophosphatemia caused by PHEX gene mutations. J Inherit Metab Dis. 2018;41(5):865-76. 10.1007/s10545-018-0147-6 PMC6133187 29460029

[9] Hardy DC, Murphy WA, Siegel BA, Reid IR, Whyte MP. X-linked hypophosphatemia in adults: prevalence of skeletal radiographic and scintigraphic features. Radiology. 1989;171(2):403-14. 10.1148/radiology.171.2.2539609 2539609

[10] Che H, Roux C, Etcheto A, Rothenbuhler A, Kamenicky P, Linglart A, et al. Impaired quality of life in adults with X-linked hypophosphatemia and skeletal symptoms. Eur J Endocrinol. 2016;174(3):325-33. 10.1530/EJE-15-0661 26783348

[11] Veilleux LN, Cheung M, Ben Amor M, Rauch F. Abnormalities in muscle density and muscle function in hypophosphatemic rickets. J Clin Endocrinol Metab. 2012;97(8):E1492-8. 10.1210/jc.2012-1336 22639288

[12] Carpenter TO, Whyte MP, Imel EA, Boot AM, Hogler W, Linglart A, et al. Burosumab Therapy in Children with X-Linked Hypophosphatemia. N Engl J Med. 2018;378:1987-98. 10.1056/NEJMoa1714641 29791829

[13] Insogna KL, Briot K, Imel EA, Kamenický P, Ruppe MD, Portale AA, et al. A Randomized, Double-Blind, Placebo-Controlled, Phase 3 Trial Evaluating the Efficacy of Burosumab, an Anti-FGF23 Antibody, in Adults With X-Linked Hypophosphatemia: Week 24 Primary Analysis. J Bone Miner Res. 2018;33(8):1383-93. 10.1002/jbmr.3475 29947083

[14] Colares Neto GP, Ide Yamauchi F, Hueb Baroni R, Bianchi MA, Gomes AC, Chammas MC, et al. Nephrocalcinosis and Nephrolithiasis in X-Linked Hypophosphatemic Rickets: Diagnostic Imaging and Risk Factors. J Endocr Soc. 2019;3(5):1053-61. 10.1210/js.2018-00338 PMC6497922 31065622

[15] Beck-Nielsen SS, Brock-Jacobsen B, Gram J, Brixen K, Jensen TK. Incidence and prevalence of nutritional and hereditary rickets in southern Denmark. Eur J Endocrinol. 2009;160(3):491-7. 10.1530/EJE-08-0818 19095780

[16] Rafaelsen S, Johansson S, Ræder H, Bjerknes R. Hereditary hypophosphatemia in Norway: a retrospective population-based study of genotypes, phenotypes, and treatment complications. Eur J Endocrinol. 2016;174(2):125-36. 10.1530/EJE-15-0515 PMC4674593 26543054

[17] Endo I, Fukumoto S, Ozono K, Namba N, Inoue D, Okazaki R, et al. Nationwide survey of fibroblast growth factor 23 (FGF23)-related hypophosphatemic diseases in Japan: prevalence, biochemical data and treatment. Endocr J. 2015;62(9):811-6. 10.1507/endocrj.EJ15-0275 26135520

[18] Instituto Brasileiro de Geografia e Estatística (IBGE). IBGE Journal. Available from: https://www.ibge.gov.br/cidades-e-estados/pr/ . Access on: Dec. 2018.

[19] Beck-Nielsen SS, Jensen TK, Gram J, Brixen K, Brock-Jacobsen B. Nutritional rickets in Denmark: a retrospective review of children's medical records from 1985 to 2005. Eur J Pediatr. 2009;168(8):941-9. 10.1007/s00431-008-0864-1 18985384

[20] Emma F, Cappa M, Antoniazzi F, Bianchi ML, Chiodini I, Vainicher CE, et al. X-linked hypophosphatemic rickets: an Italian experts’ opinion survey. Ital J Pediatr. 2019;45(1):67. 10.1186/s13052-019-0654-6 PMC6545008 31151476

[21] Verge CF, Lam A, Simpson JM, Cowell CT, Howard NJ, Silink M. Effects of therapy in X-linked hypophosphatemic rickets. N Engl J Med. 1991;325(26):1843-8. 10.1056/NEJM199112263252604 1660098

[22] Vaisbich MH, Koch VH. Hypophosphatemic rickets: results of a long-term follow-up. Pediatr Nephrol. 2006;21(2):230-4. 10.1007/s00467-005-2077-4 16252097

[23] Ha YS, Tchey DU, Kang HW, Kim YJ, Yun SJ, Lee SC, et al. Phosphaturia as a promising predictor of recurrent stone formation in patients with urolithiasis. Korean J Urol. 2010;51(1):54-9. 10.4111/kju.2010.51.1.54 PMC2855459 20414412

[24] Skrinar A, Dvorak-Ewell M, Evins A, Macica C, Linglart A, Imel EA, et al. The Lifelong Impact of X-Linked Hypophosphatemia: Results From a Burden of Disease Survey. J Endocr Soc. 2019;3(7):1321-34. 10.1210/js.2018-00365 PMC6595532 31259293

